# Rare Presentation of Deep Vein Thrombosis and Submassive Pulmonary Emboli Due to Hypercoagulable State With Supratherapeutic Anticoagulation

**DOI:** 10.7759/cureus.17300

**Published:** 2021-08-19

**Authors:** Mark D Rivera-Morales, Jesse C Wu, Larissa Dub, Latha Ganti

**Affiliations:** 1 Emergency Medicine, University of Central Florida Hospital Corporation of America (HCA) Healthcare Graduate Medical Education Consortium Emergency Medicine Residency Program of Greater Orlando, Orlando, USA; 2 Emergency Medicine, Osceola Regional Medical Center, Kissimmee, USA; 3 Emergency Medicine, University of Central Florida College of Medicine, Orlando, USA; 4 Emergency Medicine, Envision Physician Services, Plantation, USA; 5 Emergency Medicine, University of Central Florida Hospital Corporation of America (HCA) Healthcare Graduate Medical Education Consortium Emergency Medicine Residency Program of Greater Orlando, Olrando, USA

**Keywords:** supratherapeutic inr, thromboembolism, deep venous thrombosis, pulmonary embolism, hypercoagulable

## Abstract

We present a case of an elderly male with multiple co-morbidities, including atrial fibrillation on warfarin and recently diagnosed left lower extremity deep vein thrombosis (DVT), who presented to the emergency department for dyspnea. He was found to be hypoxic and mildly hypotensive. He was diagnosed with submassive pulmonary emboli (PE) despite having a supratherapeutic international normalized ratio (INR). In this case report, the clinical presentation, diagnostic workup, and management of this patient are discussed.

## Introduction

Approximately 200,000 people are diagnosed with pulmonary emboli (PE) each year in the United States, and most are derived from a lower extremity deep vein thrombosis (DVT). About 4-5% of the cases of PE will develop shock and 45% of those cases with shock will die [1]. Virchow’s triad (stasis, vascular injury, and hypercoagulability) is classically taught as the origin of thromboembolism, including PE. Common risk factors for PE include recent major surgery, trauma, prolonged immobility, malignancy, chronic inflammatory or autoimmune disorder, infection, congenital hypercoagulable state (e.g., factor V Leiden mutation), old age, pregnancy, and oral contraceptive use. Patients with PE commonly present with dyspnea on exertion, pleuritic chest pain, cough, orthopnea, hemoptysis, and leg pain or swelling. Upon examination, these patients may have tachypnea, tachycardia, and unilateral calf swelling or tenderness. Electrocardiogram (ECG) and chest radiograph (CXR) findings are often nonspecific, with sinus tachycardia being the most common abnormal ECG finding and Westermark sign and Hampton’s hump as the classic CXR findings in PE.

Several decision tools are available to risk stratify patients suspected to have PE, such as the Well’s criteria and Revised Geneva Score. In combination with the d-dimer assay, these tools are useful in ruling out patients with low to moderate risk for PE. Computed tomography pulmonary angiography (CTPA) has been the gold standard in diagnosing PE with ventilation-perfusion (V/Q) scan reserved for those patients who have impaired renal function or are allergic to contrast. In addition, bedside ultrasound has also been a useful adjunct in diagnosing PE, especially at identifying those patients with significant right ventricular (RV) dysfunction. Patients with low-risk PE determined by the PE Severity Index (PESI) can be safely discharged with direct oral anticoagulation without an increase in mortality, recurrent PE, or major bleeding [2]. For stable patients who require admission, low molecular weight heparin is preferred due to its low risk of bleeding, death, and lower cost [3].

Submassive PE is defined as patients with systolic blood pressure >90 mmHg but with signs of RV dysfunction either on ultrasonography or as evidenced by elevated troponin or B-natriuretic peptide (BNP). The cornerstone of treatment is to initiate anticoagulation and hemodynamic support with supplemental oxygenation to maintain normoxemia, judicious fluid support, vasopressor to support blood pressure, and pulmonary vasodilator to improve pulmonary vascular resistance [4]. In massive PE (patients with hemodynamic decompensation), thrombolysis has been deemed an effective treatment if there are no contraindications [5,6]. The benefits of thrombolysis in submassive are less clear because there is still a risk of intracranial hemorrhage (ICH) [7,8]. Catheter-directed thrombolysis may be an expensive option for patients who are 65 years or older with increased risk of ICH or major bleeding at the expense of increased intensive care unit (ICU) length of stay (LOS) [9,10]. In patients with massive PE, surgical embolectomy may be an option if the patient has a contraindication to thrombolysis or is unresponsive to thrombolysis [11]. Finally, extracorporeal membrane oxygenation (ECMO) may be the last resort for patients with massive PE who have refractory shock, cardiac arrest, or contraindication for thrombolysis or surgical embolectomy [12].

## Case presentation

An 86-year-old male presented to our emergency department (ED) with complaints of shortness of breath (SOB) and dyspnea on minimal exertion. The patient had a past medical history significant for chronic obstructive pulmonary disease (COPD), congestive heart failure with preserved ejection fraction, and atrial fibrillation on anticoagulation with warfarin, hypertension, dyslipidemia, a cerebrovascular accident without any residual deficits, seizures, benign prostatic hyperplasia (BPH), and a recently diagnosed left lower extremity DVT. The patient presented via EMS to our emergency department from home as a “CPAP alert.” The patient was placed on CPAP en route to the hospital because, at home, he had a SpO_2_ of 77% on room air and was notably short of breath. The patient’s respiratory status rapidly improved with CPAP and upon arrival to the ED, he was placed on supplemental oxygen via nasal cannula at 4 liters per minute and was saturating well with SpO_2_ of 98%. Upon further history, the patient endorsed dyspnea, in addition to left leg pain and swelling for the past month. He did not seek earlier medical care secondary to other issues at home. He also reported intermittent abdominal pain associated with eating as well as watery diarrhea for the past month. He denied fever, chest pain, nausea, vomiting, syncope, or any other complaints on review of systems.

**Figure 1:**
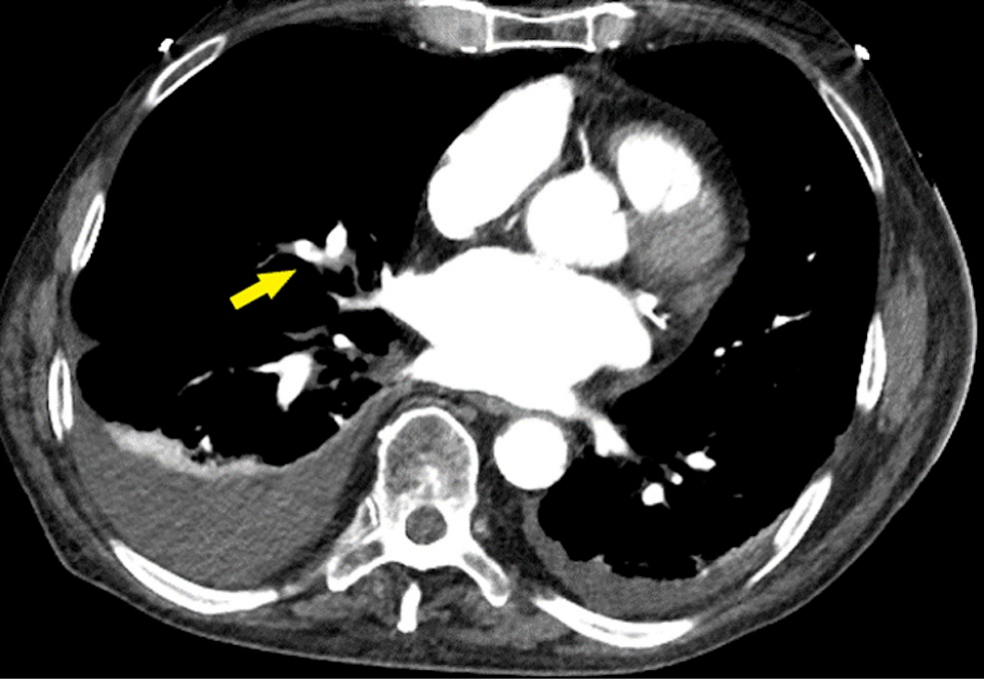
Computed tomography angiography showing pulmonary embolism, identified in the right middle lobe pulmonary arterial segments (yellow arrow).

**Figure 2:**
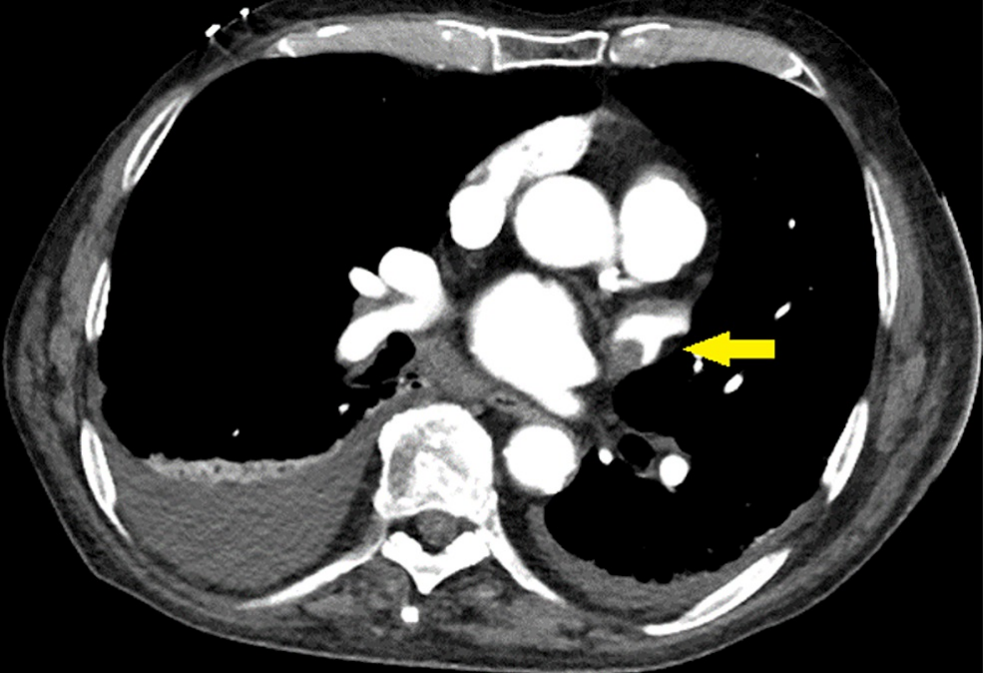
Computed tomography pulmonary angiography showing a left atrial appendage thrombus (yellow arrow).

In the ED, the patient was found to have a left lower extremity DVT, bilateral pulmonary embolism diagnosed by CTPA, as well as a left atrial appendage thrombus (Figures 1-2).

Labs were significant for a supratherapeutic INR of 7.5 and an elevated activated partial thromboplastin time (aPTT) of 54.7 seconds. Also, N-terminal proBNP levels were elevated at 2831 pg/ml, troponin-I was not elevated. Twelve-lead ECG showed atrial fibrillation with a normal rate. A cardiologist was consulted, who recommended initiation of subcutaneous enoxaparin despite a supratherapeutic INR and vitamin K reversal with oral phytonadione. The plan included transitioning the patient to a direct oral anticoagulant (DOAC), such as apixaban, prior to discharge. Hematology was consulted to weigh in on the evaluation of this hypercoagulable state despite being appropriately anticoagulated with warfarin. The patient was admitted to the cardiovascular ICU in stable but guarded condition.

## Discussion

PE is a potentially life-threatening diagnosis that cannot be missed in the ED, especially when a patient with risk factors presents with signs and symptoms that suggest PE. It is well known that PE can lead to hypoxemia via V/Q mismatch, which can increase pulmonary vascular resistance and subsequently further impair RV function and cardiac output [13]. Therefore, it is important to provide supplemental oxygenation, such as a nasal cannula or non-invasive ventilation, in patients who are hypoxic. However, PE patients, especially those with submassive and massive PE, are extremely preload dependent. Thus, it is paramount to try to avoid any interventions that could increase intrathoracic pressure that further depresses the preload, such as endotracheal intubation. In patients who are already hypotensive or have hemodynamic decompensation, intubation can lead to cardiac arrest [14]. Fortunately, this patient only required nasal cannula oxygenation to maintain his oxygen saturation. It is remarkable that this patient was well appearing without obvious respiratory distress despite his initial hypoxemia that required non-invasive ventilation. Clinicians must always remain vigilant because patients with submassive PE can decompensate at any time.

Intravenous (IV) fluid administration is the traditional teaching to improve preload. However, fluid loading must be judicious in the CHF patient to prevent fluid overloading. In addition, there are some studies that suggest IV fluid administration may actually worsen RV function and impair cardiac output in patients who already have severe RV dysfunction [15]. In this case, volume off-loading with diuretics has been advocated to improve RV function in patients with submassive PE [16]. In patients with acute hemodynamic decompensation, vasopressors such as norepinephrine should be administered to improve cardiac output and coronary perfusion, especially if the patients have contraindications to thrombolysis [4,17]. More recently, pulmonary vasodilators, such as inhaled nitric oxide, have been demonstrated to decrease pulmonary vascular resistance, RV afterload, and improve V/Q mismatch [18]. It is a potential intervention in patients with shock refractory to vasopressor or thrombolysis. Even though our patient was borderline hypotensive, he was able to maintain his SBP >90 mmHg and has no signs of RV strain on bedside cardiac ultrasound. Therefore, vasopressor and thrombolysis were not necessary. Even if this patient were acutely decompensating, thrombolysis would have been contraindicated since he was on warfarin.

It is puzzling that this patient developed a DVT and bilateral PE despite being on warfarin with a supratherapeutic INR. However, cases of PE while on therapeutic or supratherapeutic anticoagulation have been described in the literature [19]. Perhaps this patient had an undiagnosed underlying malignancy, as evidenced by the pulmonary nodules and enlarged retroperitoneal lymph nodes on CT, which contributed to his hypercoagulable state. There are multiple studies demonstrating that low molecular weight heparin and direct factor Xa inhibitors are superior to warfarin in terms of preventing recurrent venous thromboembolism and decreasing the incidence of major bleeding [20]. Therefore, it makes sense to discontinue warfarin on this patient and continue this patient on low molecular weight heparin or apixaban upon discharge.

**Figure 3:**
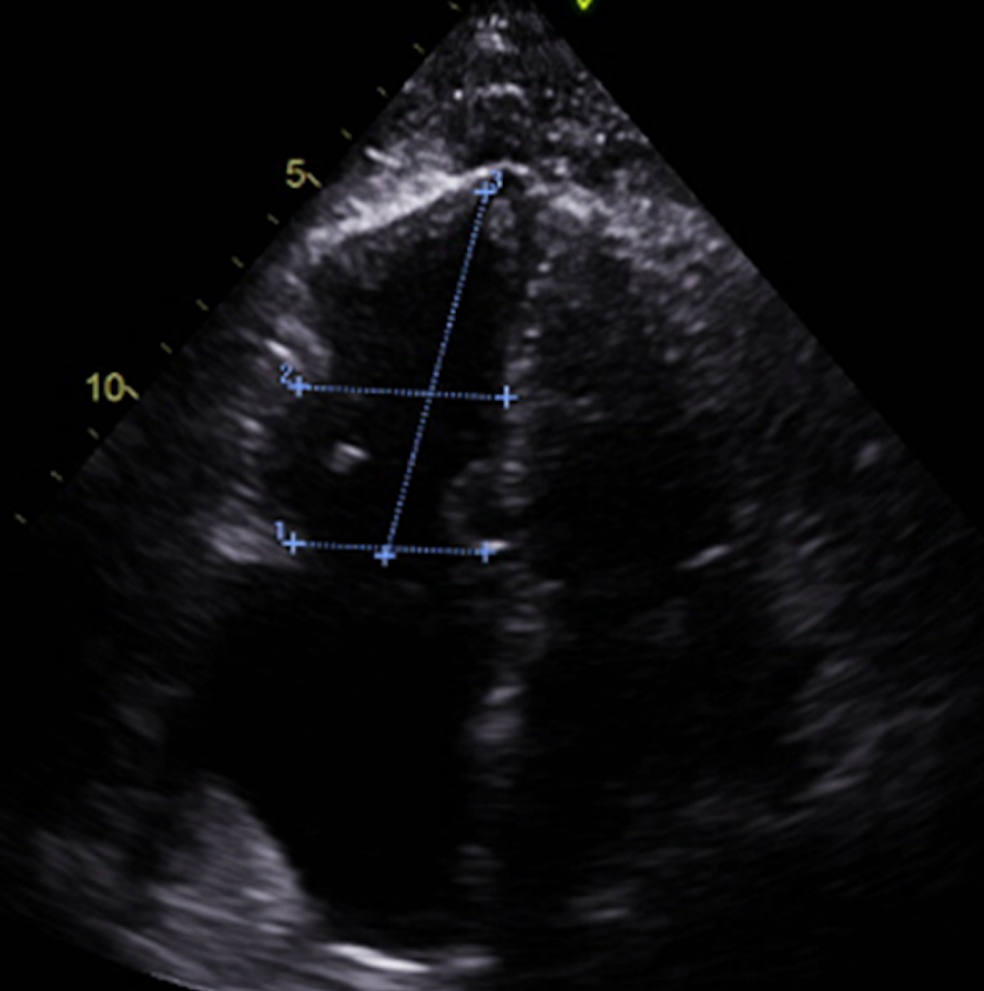
Two-dimensional echocardiogram showing RV dilation. A4CH RV diameter was 3.7 cm suggestive of RV dysfunction. In comparison, the A4CH LV diameter was 3.1 cm. RV: right ventricle, A4CH: apical 4 chambers, LV: left ventricle.

It is suspected that our patient likely has an undiagnosed malignancy as evidenced by the two pulmonary nodules, enlarged retroperitoneal lymph nodes, and small volume ascites found incidentally on CT imaging. This was believed to have contributed to his hypercoagulable state. The 2D echocardiogram done after admission did show evidence of mild RV dilation suggestive of RV dysfunction (Figure 3).

The patient remained hemodynamically stable during his stay in the hospital. He subsequently was converted to oral apixaban and warfarin was discontinued. After discussion with the patient and family, the patient was discharged home with hospice care and outpatient follow-up with hematology was recommended.

## Conclusions

Pulmonary embolism is a life-threatening diagnosis that should be considered in any patient who presents to the emergency department with symptoms of dyspnea, chest pain, palpitations, syncope, or unilateral leg swelling. Risk factors should be immediately reviewed and all presenting symptoms should be evaluated. Clinicians must balance the patient's delicate respiratory state while closely monitoring the patient's oxygen demands and respiratory drive. Fluid resuscitation must be judicious and close hemodynamic monitoring must be utilized in patients with massive and submassive PE. Early vasopressor support or thrombolysis should be considered in patients with no contraindications who present with refractory hypotension and hemodynamic instability. Finally, involving the appropriate subspecialists in patients who continue to experience thrombotic events despite therapeutic or supratherapeutic anticoagulation should be considered.
